# Shape matters: the pitfalls of analyzing mesophyll anatomy

**DOI:** 10.1111/nph.16360

**Published:** 2019-12-28

**Authors:** Guillaume Théroux‐Rancourt, Klara Voggeneder, Danny Tholen

**Affiliations:** ^1^ Institute of Botany University of Natural Resources and Life Sciences Vienna 1180 Austria

**Keywords:** leaf anatomy, mesophyll, microCT, serial sectioning, stereology, structure–function relations

## Abstract

This article is a Commentary on https://doi.org/10.1111/nph.16219.

It is well‐established that anatomy facilitates and constrains leaf function (Oguchi *et al.*, 2018). Such research on structure–function relationships emerged from a fascination with plant cell shapes that botanists have displayed for centuries (e.g. Haberlandt, 1904). While some leaf cells have mesmerizing anatomy, such as the pavement cells or trichomes of the epidermis, others, like the photosynthesizing mesophyll cells, may seem less alluring. In a cross‐section through the leaf, the palisade tissue is visible as one or more layers of rectangles, whereas the spongy cells have an elliptical or circular shape. Indeed, palisade cells have sometimes been simplified to cylinders and the spongy cells to spheres in textbooks discussing structure–function relationships (e.g. Nobel, 2009). This may have led to the misconception that spongy cells are typically spherical (e.g. Martin & Hine, 2015), even though both classical (Haberlandt, 1904) and more modern literature (Ivanova & P'yankov, 2002; Oguchi *et al.*, 2018) show clearly that mesophyll cells are often lobed, and spongy cells in particular are often irregularly shaped.

‘We strongly urge researchers to be aware of the limitations of the methods used to analyze anatomy …’

In this issue of *New Phytologist*, Harwood *et al.* (2020; pp. 2567–2578) use serial block‐face scanning electron microscopy (SBF‐SEM) to highlight this perception issue. Serial sectioning, the act of making consecutive microscopic sections to capture the real three‐dimensional (3D) nature of the cells in the leaf, would allow for more accurate estimates of volume and surface area. Such estimates are important in the context of understanding gas diffusion, water transport, light reception, and mechanical properties of the plant leaf (Oguchi *et al.*, 2018). Manual serial sectioning of mesophyll tissue for electron microscopy is very labor‐intensive (Zellnig *et al.*, 2004), allowing only small parts of the mesophyll cells to be examined. Harwood *et al.* show that the block‐face scanning technique, where the sectioning is automated, makes it now less labor‐demanding to acquire larger volumes of the leaf in three dimensions.

Harwood *et al.* raise the important point that leaf anatomy is commonly studied in two dimensions. They rightly argue that simple geometrical models do not represent the true shape of the mesophyll cells, generally leading to underestimation of volume and surface area.

However, in this Commentary, we would like to point out an additional important drawback of the use of two‐dimensional (2D) sections: a single random cross‐section of a 2D object does not allow for the extraction of correct quantitative information on surface area or volume. Assuming an isotropic tissue (i.e. uniform in all directions), the average volume of cells, organelles, or airspaces can be reasonably estimated from a single cross‐section through a tissue, because the planar area of a structure is a sampling of its volume. However, the curvature of the surface cannot be observed in a single slice and estimates of surface area require curvature corrections that are prone to bias because they rely on assumptions regarding the shape of the cells or organelles (e.g. Thain, 1983; Théroux‐Rancourt *et al.*, 2017). If the object of interest is a single cell or organelle, it becomes even more difficult to obtain unbiased estimates of volume and surface area. A number of approaches, discussed in Harwood *et al.*, have been used to quantify cell or organelle volume and surface area by using exact or approximate estimates of volumes and surface areas for spheres, cylinders, capsules, or ellipsoids. These approaches not only rely on questionable assumptions on the cell shape as highlighted by Harwood *et al.*, but they cannot be applied on single sections because they usually underestimate the true dimensions of the structure under investigation (see geometrical example in Box 1).

Box 1 1 Cross sections taken at random through a sphere will result in circles that on average have a radius

(1) $$r_{c}=\int_{-R_{s}}^{R_{s}}\sqrt{R_{s}^{2}-h^{2}}dh/\left(2R_{s}\right)=\pi R_{s}/4\approx 0.79R_{s}\text{,}$$

where *R*_s_ is the radius of the sphere. Therefore, calculating the volume of the sphere as $V_{s}=\frac{4}{3}\pi r_{c}^{3}$ and its surface area as $S_{s}=4\pi r_{c}^{2}$ will underestimate the true values by 52% and 38%, respectively.

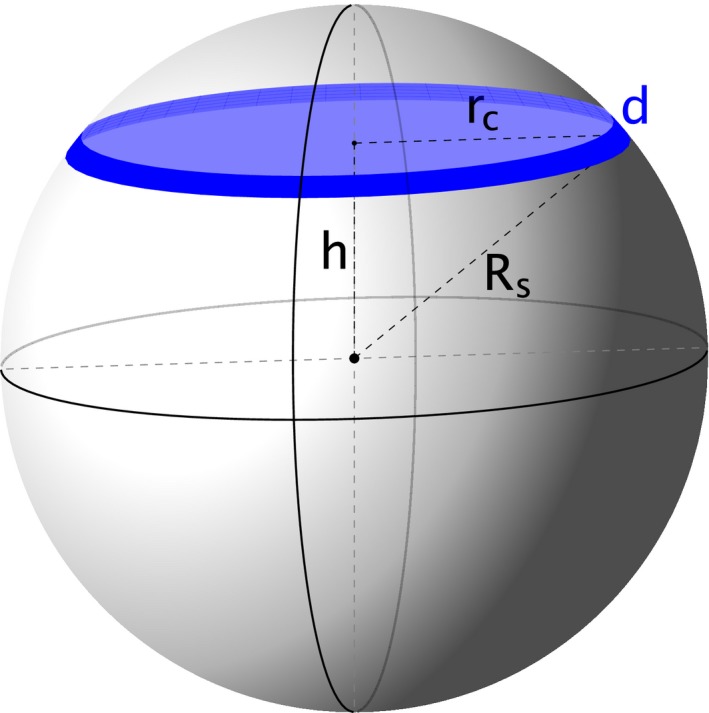

## Available in stereo(logy)

Considerations such as the earlier have led to the development of stereological methods, that is, methods that extract quantitative information on anatomical structure from partial, usually lower dimensional, data. Such methods have been developed before 3D imaging methods (e.g. confocal microscopy, tomography) became widely available. Perhaps as a result of their more laborious nature, stereological approaches have only rarely been applied to quantitative analysis of plant anatomy (reviewed in Kubínová *et al.*, 2017). Design‐based stereological methods aim to provide estimates of, for example, volume and surface area that are independent of size, shape, orientation, or distribution of the material. Such methods typically involve taking 2D samples (images) from a 3D structure according to a given sampling design and using statistical principles to estimate 3D properties such as volume and surface area (Kubínová *et al.*, 2017). The greatest advantage of such techniques for analyzing the leaf mesophyll is that no assumptions on cell shapes are necessary.

**Figure 1:**
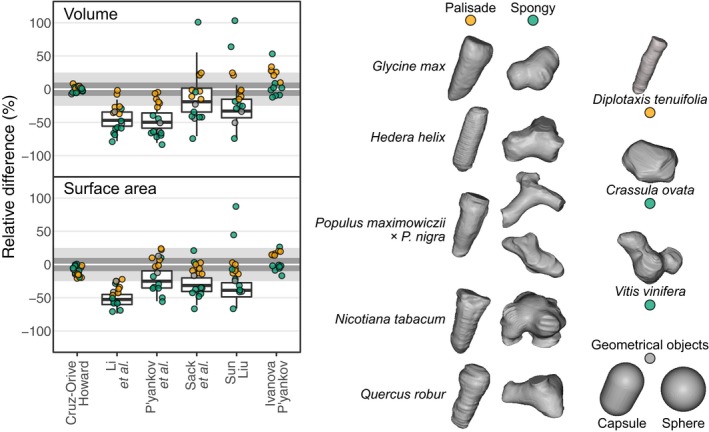
Relative difference of three‐dimensional (3D) values for volume and surface area of whole cells extracted from microCT imaging to values estimated by stereology (Cruz‐Orive & Howard, 1995), the geometrical methods used by Harwood *et al*. (2020) in their *New Phytologist* article in this issue (pp. 2567–2578) (P'yankov *et al.*, 1999; Sun & Liu, 2003; Li *et al.*, 2013; Sack *et al.*, 2013), as well as for the projection method of Ivanova & P'yankov (2002). Except for the projection method, 20 random but evenly spaced two‐dimensional (2D) sections were analyzed. We extracted single palisade (orange) and spongy (green) cells from microCT scans of eight species, measured volume and surface areas using automated image analysis (Doube *et al.*, 2010), and plotted the relative differences compared to the other methods on top of the values for the chickpea mesophyll cells from Harwood *et al.*, relative to their serial block‐face scanning electron microscopy (SBF‐SEM) 3D estimates (boxplots; see Supporting Information Methods S1). The dark gray and light areas highlight the area within 10% and 25% from the 3D value. Across the species studied, palisade cells were elongated, but did not have a typical capsule‐like shape. Instead, the diameter of these cells generally decreased somewhat towards the bottom, at the intersection between palisade and spongy mesophyll. Most spongy cells had irregular shapes, often trilobed, except for in *Crassula ovata,* which presented the typical inflated shape of succulent cells, although not perfectly regular. *Populus maximowiczii* × *P. nigra* had spongy cells with very long protrusions near the abaxial epidermis, and cells with shorter protrusions closer to the palisade mesophyll. For comparison, two mathematical shapes (gray) used in some of the geometrical methods are shown. Cell shapes are available online at doi: https://doi.org/10.6084/m9.figshare.11282066.

Harwood *et al.* estimated volume and surface of their 3D cell reconstructions and compared these with four different methods that rely on assumptions regarding the shape of the cells. We compared these same geometrical models with a stereological method using cells extracted from microCT scans acquired at the TOMCAT beamline of the Swiss Light Source (Paul Scherrer Institute, Villigen, Switzerland) as a way to expand Harwood *et al.*’s dataset, although at a lower magnification compared to SBF‐SEM (Fig. 1; Supporting Information Dataset S1; cell shapes are available at doi: https://doi.org/10.6084/m9.figshare.11282066). The results clearly show that using geometrical models for estimating volumes and surfaces results in large biases. Generally, the volume and surface area of palisade cells, being more regular and cylindrical (Fig. 1), are underestimated between 10% and 25% relative to the 3D estimates. The values for spongy cells deviate even more from the 3D values as their irregular shape differs significantly from the sphere or ellipsoids assumed by the geometrical methods (Fig. 1). We also included mathematical objects such as spheres and capsules in the analysis to show that even when the cell shape matches the assumptions made by the geometrical methods, the estimated volume and surface area are still underestimated for the reasons mentioned earlier (Box 1).

Estimating volumes and surfaces from stacks of images typically involves segmenting the tissue before volume and surface areas can be assessed by automated image analysis (Théroux‐Rancourt *et al.*, 2017; Harwood *et al.*). However, a common technique in stereology is placing a grid over the 2D images and intersections of this grid with the structure of interest are counted to estimate volume and surface area with a statistical model. Several parallel sections through the tissue allow for estimation of the volume using interpolation and Cavalieri's principle (e.g. Cruz‐Orive & Howard, 1995). Estimating the surface area is more complicated as the sections would need to be cut isotropically (randomly over all directions) to account for curvature. Instead, Cruz‐Orive & Howard (1995) present a method to estimate surface area that involves sampling an anisotropic stack of sections with a 3D grid. Fig. 1 shows that this approach leads to estimates very close to the 3D values based on segmented images.

As an alternative to stereology, Ivanova & P'yankov (2002) described a method based on projections of cells. Fig. 1 shows that this method is better than most geometrical approximations, especially for the spongy cells with protrusions. However, an important drawback is that the approach requires macerating the leaf material to obtain the isolated cells needed for making the projections.

## What can be done

Harwood *et al.* issued a warning to users of methods based on analyzing just one or a few sections per replica. We reiterate and highlight this warning here by expanding their analysis to more species. We strongly urge researchers to be aware of the limitations of the methods used to analyze anatomy, and to clearly state them in their research papers. Are only 3D methods acceptable? In our opinion not, as purely 3D methods rely on equipment that remains difficult to access (synchrotron‐based microCT) or still lacks wide availability (SBF–SEM). Serial sections provide a good alternative and can easily be made from embedded material using widely available microtomes. After microscopy, images can be aligned, segmented, and 3D representations can be made that allow for estimation of volume or surface area. Such stacks can also easily be obtained using optical sectioning with a laser confocal microscope. Recent approaches show that scans of up to 100 µm deep in the leaf are possible (Littlejohn *et al.*, 2014).

The additional work of making serial sections and subsequent image segmentation may seem daunting, but if a complete 3D reconstruction of the anatomy is not required, stereological approaches on a few sections can be used to estimate volumes, surfaces, and cell number with far less effort. We show here that 20 random but evenly spaced sections allow for accurate estimates of cell volume and surface area. Moreover, our dataset shows that even with as few as five sections variation increases, but on average the estimates are still more accurate than those based on 20 sections with the geometrical methods shown in Fig. 1. Averaging volume and surface area estimates of several cells would be a suitable alternative for most applications.

Although we emphasize that accurate 2D approaches are available to estimate surface and volume, state‐of‐the‐art methods, like the SBF‐SEM used by Harwood *et al.*, provide grounding for 3D physico‐chemical models of plant physiology that help understand light absorption, water flow, gas diffusion, and biomechanics. In addition, such methods push the boundaries in observing the complexity and variation of cell shapes in plant leaves. As in all biological systems, diversity is key, and reducing mesophyll to a few idealized shapes obscures our understanding of how cells affect the structure of a leaf and its function: shape matters.

## Supporting information


**Dataset S1** Volume and surface area measured in three‐dimensions (3D) and computed from the different estimation methods for each cell and geometrical object presented in Fig. 1.

**Methods S1** Description of the methods used to extract cells from microCT images and to estimate volume and surface area using the different methods presented in Fig. 1.
Please note: Wiley Blackwell are not responsible for the content or functionality of any Supporting Information supplied by the authors. Any queries (other than missing material) should be directed to the *New Phytologist* Central Office.
