# Supplementary material for: Shape matters: the pitfalls of analyzing mesophyll anatomy
Source: New Phytol. 2019 Dec 28;225(6):2239–42. doi: 10.1111/nph.16360 (PMC7160601; doi:10.1111/nph.16360)
Supplement: Supplementary file 2 — Methods S1 Description of the methods used to extract cells from microCT images and to estimate volume and surface area using the different methods presented in Fig. 1. Please note: Wiley Blackwell are not responsible for the content or functionality of any Supporting Information supplied by the authors. Any queries (other than missing material) should be directed to the New Phytologist Central Office. [file NPH-225-2239-s002.pdf]

Supplementary methods S1

microCT imaging and cell segmentation

Potted plants (*Glycine max*, *Nicotiana tabacum*, *Diplotaxis tenuifolia*, *Vitis vinifera*) and cut stems (*Populus maximowiczi* x *P. nigra*, *Quercus robur*, *Hedera helix*; wrapped in wet paper towels and stored in plastic bags) were brought to the TOMCAT tomography beamline of the Swiss Light Source (Paul Scherrer Institute, Villigen, Switzerland). Leaves were prepared as in Théroux-Rancourt *et al.* (2017), where a leaf strip ~2 mm wide and ~15 mm long was cut between the midrib and the edge of the lamina, avoiding major veins. It was immediately wrapped and sealed between layers of Kapton (polyimide) tape. Leaves were placed in a Styrofoam holder placed ~20-40 mm away from the detector and centered on the microCT stage to image a region slightly above the Styrofoam. 1,800 projections of 100 ms exposure were acquired at 21 keV using the 20x or 40x objective, resulting in final pixel sizes of 0.35 or 0.1625 µm, respectively. Scans were reconstructed with the (Dowd *et al.*, 1999) and phase retrieval (Paganin *et al.*, 2002) reconstruction methods using the in-house platform.

On the reconstructed scans in ImageJ (Schneider *et al.*, 2012), a threshold was applied, i.e. cells were labelled one color and airspace another, using the reconstruction method that allowed to better distinguish and segment the contours of the cells (the gridrec reconstruction was most often used). Using both the thresholded stack and original stack side-by-side, a cell was manually segmented on the paradermal plane by delimiting its edges and filling its interior with a different color than used for neighboring cells and airspace. This was repeated for all slices containing the cell. The cell was then extracted from the whole leaf stack by thresholding for its specific color. To correct for segmentation errors occurring when manually segmenting slice-by-slice, the cell was examined with ImageJ's 3D viewer to detect too large areas segmented compared to neighboring regions of the cell (e.g. resulting from identifying cell regions being thought to belong to the cell of interest but actually belonging to another cell). Slices with mistakes were corrected, and cells were finally smoothed out using one or multiple passes of a 3D Gaussian blur of 2-pixels radius. Cell stacks are available online (doi:10.6084/m9.figshare.11282066)

3D image analysis

Volume and surface area (SA) were measured using the *Analyze Particles* function of the ImageJ BoneJ plugin (Doube *et al.*, 2010) using a surface resampling of 2. This resampling gave volumes and SA closest to the values from the mathematical equations for 3D stack of spheres. A lower resampling, i.e. a finer meshing of the surface, potentially overestimates values compared to the true mathematical values because of the rasterization of the surface.

2D image analysis

For stereological analysis of the stacks, we used the Grid Cycloid Arc ImageJ plugin (© E. Kischell & T.E. Andersen, https://imagej.nih.gov/ij/plugins/grid-cycloid-arc.html), but shifted the grid vertically on slices deeper in the stack as described in Cruz-Orive & Howard (1995) to create cycloids having the same amplitude and frequency in the *xy* and *yz* planes (see their Fig. 3). Cycloids were created on 19 to 22 slices evenly covering the whole cell. The number of intersections between the cycloids and the cell perimeter was counted, as well as the number of intersections between the grid and the cross-sectional area of the cell and the number of such intersections from the previous slice that were still intersecting with the area in the current slice. This allows for estimates of volume and SA using the equations described by Cruz-Orive & Howard (1995; see their Equations 4 and 9).

For the projection method of Ivanova & P'yankov (2002), we created a projection of the cell showing its largest surface by flattening the stack using the *Z Project* function of ImageJ. The area and perimeter of the projection were measured. Cell or protrusion diameter and length were measured to estimate the coefficients necessary for the volume and SA equations of Ivanova & P'yankov (2002; see their Equations 1 and 5 and Tables 2 and 3).

For the geometrical methods used by Harwood *et al.* (2019), i.e. the P'yankov *et al.* (1999), Sun & Liu (2003), Li *et al.* (2013), and Sack *et al.* (2013), the perimeter, area, and Feret diameter were measured in ImageJ for every slice of each cell or mathematical object oriented in their cross-sectional view (i.e. how it would be observed on an 2D cross section). Volume and SA were estimated using each geometrical method, accounting for cell type (spongy or palisade). The same slice positions as used for the Cruz-Orive & Howard (1995) method were used to compute the median volume and SA for each geometrical method (median values would

represent the area that is most commonly measured; also, mean values gave larger differences to 3D values).

The difference between the 3D and 2D value was then computed. For the data from Harwood *et al.* (2019), we computed the difference between their 3D and 2D values.

References

Cruz-Orive LM, Howard CV. **1995**. Estimation of individual feature surface area with the vertical spatial grid. *Journal of Microscopy* **178**: 146–151.

Doube M, Kłosowski MM, Arganda-Carreras I, Cordelieres FP, Dougherty RP, Jackson JS, Schmid B, Hutchinson JR, Shefelbine SJ. **2010**. BoneJ: free and extensible bone image analysis in ImageJ. *Bone* **47**: 1076–1079.

Dowd BA, Campbell GH, Marr RB, Nagarkar VV, Tipnis SV, Axe L, Siddons DP. **1999**. Developments in synchrotron x-ray computed microtomography at the National Synchrotron Light Source. In: Bonse U, ed. *SPIE's International Symposium on Optical Science, Engineering, and Instrumentation.* SPIE, 224–236.

Harwood R, Goodman E, Gudmundsdottir M, Huynh M, Musulin Q, Song M, Barbour MM. **2019**. Cell and chloroplast anatomical features are poorly estimated from 2D cross-sections. *New Phytologist.* doi: 10.1111/nph.16219.

Ivanova LA, P'yankov VI. **2002**. Structural adaptation of the leaf mesophyll to shading. *Russian Journal of Plant Physiology* **49**: 419–431.

Paganin D, Mayo SC, Gureyev TE, Miller PR, Wilkins SW. **2002**. Simultaneous phase and amplitude extraction from a single defocused image of a homogeneous object. *Journal of Microscopy* **206**: 33–40.

Pyankov VI, Kondratchuk AV, Shipley B. 1999. Leaf structure and specific leaf mass: the alpine desert plants of the Eastern Pamirs, Tadjikistan. *New Phytologist* **143**: 131–142.

Sack L, Chatelet DS, Scoffoni C. 2013. Estimating the mesophyll surface area per leaf area from leaf cell and tissue dimensions measured from transverse cross-sections. [WWW document] URL http://prometheuswiki.org/tiki-index.php?page=Estimating+the+mesophyll+surface+area+per+leaf+area+from+leaf+cell+and+tissue+dimensions+measured+from+transverse+cross-sections [accessed 1 October 2019].

Schneider CA; Rasband WS, Eliceiri KW. **2012**. NIH Image to ImageJ: 25 years of image analysis. *Nature Methods* **9**: 671–675.

Sun J, Liu D. 2003. Geometric models for calculating cell biovolume and surface area for phytoplankton. *Journal of Plankton Research* **25**: 1331–1346.

Théroux-Rancourt G, Earles JM, Gilbert ME, Zwieniecki MA, Boyce CK, McElrone AJ, Brodersen CR. **2017**. The bias of a two-dimensional view: comparing two-dimensional and three-dimensional mesophyll surface area estimates using noninvasive imaging. *New Phytologist* **215**: 1609–1622.
